# Parametric Design and Experiment on Compression Performance of Hierarchical Origami Honeycomb Structures

**DOI:** 10.3390/ma18214866

**Published:** 2025-10-24

**Authors:** Xiaohui Lu, Yong Yang, Xiang Peng

**Affiliations:** 1 School of Intelligent Manufacturing and Energy Engineering, Zhejiang University of Science and Technology, Hangzhou 310000, China; luxh@zust.edu.cn; 2 College of Mechanical Engineering, Zhejiang University of Technology, Hangzhou 310000, China; 221122020202@zjut.edu.cn

**Keywords:** hierarchical origami honeycomb structure, mechanical properties, energy absorption capacity, geometric parameter optimization, energy-absorbing material design

## Abstract

This study focuses on the energy absorption characteristics of hierarchical origami honeycomb structures. By combining experimental and numerical simulation methods, it deeply explores their mechanical properties and energy absorption potential. This research emphasizes analyzing the influence of geometric parameters (including wall thickness, folding angle, multi-layer structure design, etc.) on bearing capacity, stiffness, and energy absorption efficiency and reveals the advantages of hierarchical design in regulating gradient stiffness. The results show that the energy absorption capacity and performance of the material can be significantly improved through the reasonable optimization of geometric parameters. This research provides important theoretical support for the design of high-efficiency energy-absorbing materials and innovative solutions for energy absorption problems in related engineering applications.

## 1. Introduction

Origami is a novel design method that can produce structures with unique deformation mechanisms and shape-changing characteristics through simple folding [1,2]. As early as 1921, Dean first manufactured a honeycomb structure based on the kirigami origami method and applied for a patent [3]. Honeycomb structures are usually formed by a periodic topological distribution of unit cells, filling the internal space and interrelating with each other. They have high porosity and low mass density, thus being renowned for their excellent mechanical properties such as high specific stiffness and energy absorption capacity [4,5,6]. However, this also means they have a large volume, which becomes a great limitation in some special scenarios, especially in intelligent space deployable structures with load-bearing requirements.

Deformable origami honeycomb structures, relying on their unique geometric configurations and excellent mechanical properties, have been widely used in many engineering fields such as aerospace, biomedicine, soft robotics, and energy absorption devices [7,8]. However, once the origami structure is fixed, its mechanical properties are difficult to adjust, making it unable to adapt to variable load conditions. In most cases, the adjustable mechanical properties of thick-plate origami are incompatible with foldability and deformability. Although significant progress has been made in the design and application research of origami honeycomb structures [9], there is still a lack of a systematic strategy to realize origami honeycomb structures with adjustable stiffness through reasonable geometric design, which to a certain extent limits their further application in high-performance engineering fields.

Honeycomb structures, as energy absorption devices, have excellent energy absorption characteristics, but they encounter the problem of the uneven distribution of crushing force during deformation [10], mainly manifested as excessively high initial peak force and significant fluctuations in the platform stress stage. Origami and kirigami are effective methods for preparing lightweight honeycomb metamaterials. They change the crushing mode of the structure by introducing origami patterns and improve energy absorption performance by using folding design and geometric characteristics [11]. Zhai [12] and others studied the energy absorption characteristics of origami honeycombs during out-of-plane crushing. The numerical simulation results show that compared with traditional honeycombs, origami honeycombs exhibit a more stable folding process. In addition, due to the guiding effect of creases, the deformation mode of origami honeycombs during compression is predictable. The error range between the theoretical model based on the hyper-folding method and the simulation results is −8.55–−6.50%, verifying the accuracy of the model. Cui [13] and others studied the compression performance of a three-dimensional reentrant honeycomb structure based on periodic origami. Ye [14] and others developed a 3D self-locking thick-plate origami structure.

This paper adopts 3D printing as the manufacturing method for hierarchical origami honeycomb structures, which can construct complex three-dimensional geometric structures in a highly free manner while achieving high-precision parametric customization and integrated manufacturing [15]. This manufacturing characteristic provides great convenience for the geometric design and performance regulation of hierarchical origami honeycomb structures, making the rapid prototyping and performance optimization of complex origami honeycomb structures possible. The hierarchical deformable origami honeycomb structure designed in this paper belongs to the category of thick-panel origami, whose core feature is that the thickness of the structure cannot be ignored. Different from traditional thin-plate origami, thick-panel origami structures are composed of rigid panels rotating around predetermined hinges. It is precisely due to this structural feature that the connection relationship between panels and hinges has a decisive impact on the mechanical properties of the structure. In particular, the hinge distribution position design parameter ψ not only determines the deformation mode of the structure but also significantly affects the overall strength and stiffness characteristics of the structure.

This paper will focus on studying the inherent relationship between the geometric parameters of hierarchical origami honeycomb structures and their mechanical properties, specifically including the following three aspects: first, the influence of wall thickness, exploring the influence of different wall thicknesses on structural stiffness, strength, and deformation behavior; second, the connection position and thickness of hinges, analyzing the influence mechanism of hinge geometric parameters (hinge-to-thin-plate-thickness ratio *e*, hinge position design parameter ψ) on structural strength; and third, the gradient design of multi-layer hierarchical origami honeycomb structures. In multi-layer deformable origami honeycomb structures, by designing different geometric parameters ψ on different layers, the adjustable compression performance and gradient strength distribution of the structure are realized. Through the above research aims, it is intended to establish a systematic strategy based on geometric design to realize the planning of the stiffness and energy absorption capacity of origami honeycomb structures, providing a theoretical basis and technical support for the design and optimization of high-performance origami honeycomb structures.

## 2. Preparation Method of Hierarchical Origami Honeycomb Structure Samples by 3D Printing

**Figure 1:**
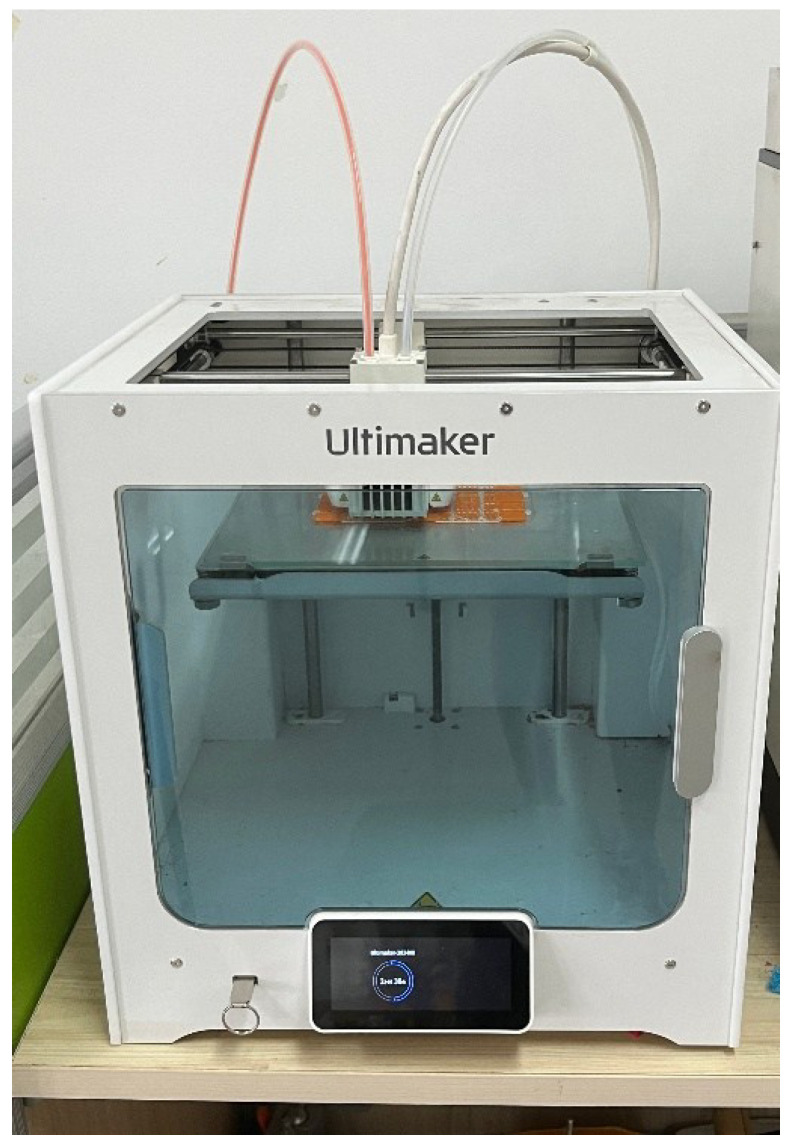
Ultimaker S3 dual-extrusion printer.

All experimental samples in this paper were prepared using an Ultimaker S3 dual-nozzle printer (Ultimaker, Geldermalsen, the Netherlands) based on fused deposition modeling technology, as shown in Figure 1. The equipment uses standard wires with a diameter of 2.85 mm, integrates a number of advanced functions to ensure printing quality, and is equipped with an intelligent active leveling system, real-time material flow monitoring module, and automatic printing parameter optimization system. These functions work together to effectively ensure a firm bonding effect between printed layers.

In the printing of each structure, all samples use the same printing parameters. Since the printing process needs to add support structures, the printing acceleration is appropriately reduced to obtain a more refined support structure, and the support structure pattern is concentric. The printing temperature directly affects the melting and fluidity of the material. Too low a temperature may lead to a poor melting effect and weak inter-layer bonding, while too high a temperature may cause the excessive melting of the material, affecting printing accuracy and details. The bottom layer temperature affects the adhesion between the first layer and the printing base plate. An appropriate bottom layer temperature helps to ensure that the first layer is firmly adhered, improves the stability of the entire printing process, and prevents warping at the bottom of the structure. Therefore, the temperatures of the nozzle and the printing platform are set to 220 °C and 50 °C, respectively.

### Finite Element Model

Finite element simulations were performed using Abaqus/Standard 2022 (Dassault Systèmes, Vélizy-Villacoublay, France) Standard to complement the experimental investigations. The geometric models were created based on the printed samples’ nominal dimensions [6] and meshed using C3D8R elements (8-node linear brick elements with reduced integration). A mesh convergence study was conducted to ensure result accuracy, with a final mesh size of approximately 0.5 mm for critical regions (hinges) and 1.0 mm for thin plates, resulting in approximately 50,000-80,000 elements per model depending on structural complexity.

The material behavior of polylactic acid (PLA) was modeled as elastic–plastic with isotropic hardening. Material properties were determined from uniaxial tensile tests of printed specimens, yielding the following: Young’s modulus *E* = 3.5 GPa; Poisson’s ratio ν = 0.36; yield stress $\sigma_{y}$ = 50 MPa; and tangent modulus $E_{t}$ = 0.1 GPa. The plastic behavior was defined using a multilinear stress–strain curve obtained from the tensile tests.

Boundary conditions were applied to simulate the experimental compression setup: the bottom surface was fully constrained (fixed in all directions), while uniform displacement was applied to the top surface in the vertical direction at a rate of 5 mm/min, consistent with the experiments. Friction between the structure and compression platens was modeled using a Coulomb friction coefficient of 0.3. The nonlinear analysis employed the Newton–Raphson method with automatic time stepping to capture the post-buckling behavior and large deformations.

To enhance the readability of the paper, a Table 1 of variable abbreviations is provided.

## 3. Definition of Mechanical Performance Metrics

To evaluate the compression performance of the hierarchical origami honeycomb structures, several key mechanical metrics are defined as follows: Stiffness: The structural stiffness *K* is calculated from the initial linear elastic region of the force–displacement curve:

(1) $$K=\frac{F}{A\cdot \varepsilon }$$

where *F* is the applied force, *A* is the cross-sectional area, and ε is the compressive strain. Specific Energy Absorption (SEA): SEA represents the energy absorbed per unit mass during compression, calculated as follows:

(2) $$\mathrm{SEA}=\frac{\int_{0}^{\delta }F\left(x\right)dx}{m}$$

where δ is the compression displacement, F(x) is the force as a function of displacement, and *m* is the mass of the specimen. Initial Peak Force: The initial peak force is the maximum force reached during the first peak in the force–displacement curve, representing the onset of structural collapse or significant plastic deformation.

## 4. Influence of Wall Thickness on Compression Performance of Hierarchical Origami Honeycomb Structures

Wall thickness has a significant impact on the strength of honeycomb structures [16], and it is considered one of the important factors that has a great impact on structural performance in structures with the same unit shape. The main research purpose of this section is to clarify the relationship between unit wall thickness and structural performance by studying the influence of different wall thicknesses on the stiffness and energy absorption of hierarchical origami honeycomb structures under the same unit structure so as to select an appropriate unit wall thickness to meet the performance requirements and realize the lightweight design of hierarchical origami honeycomb structures.

In different hierarchical origami honeycomb structures, the overall thickness of the structure is mainly changed by adjusting the d value, g value, and t value of the structure. In this section, several hierarchical deformable origami honeycomb structures with different thicknesses are designed, as shown in Table 2. The structures with different thicknesses are named HOH1 to HOH3. Considering that the diameter of the printer nozzle in the sample manufacturing process is 0.4 mm and the layer thickness is 0.2 mm, this paper selects a g value wall thickness spacing of 0.6 mm as a variable, and the d value spacing affecting the hinge thickness is 0.4 mm.

**Figure 2:**
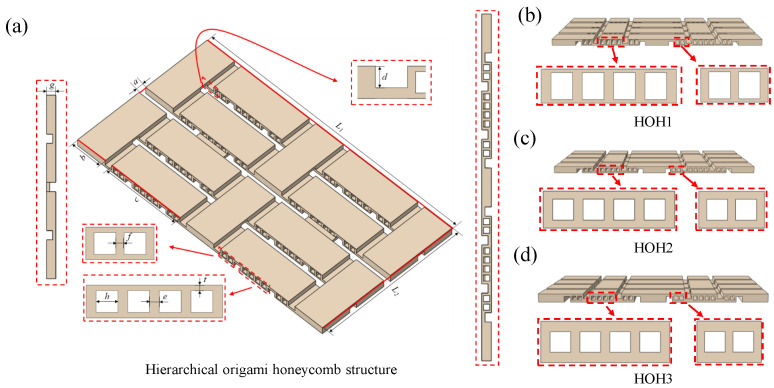
Schematic diagram of hierarchical origami honeycomb structures with different wall thicknesses. (**a**) Schematic diagram of HOH1 structure. (**b**) Schematic diagram of HOH2 structure. (**c**) Schematic diagram of HOH3 structure. (**d**) Hierarchical origami honeycomb structure.

The two-dimensional structures before deformation were produced according to the preparation method in Section 2. In the preparation stage of the printing process, the slicing software Ultimaker Cura 4.12.0 was used to slice each sample, and the weight of materials and printing time required for each sample preparation process were exported and are listed in Table 2. According to the thermal deformation test process in the team’s previous research [17], each sample was heated in turn, and an external load was applied to convert them into corresponding three-dimensional structures, which were named HOH1-D to HOH3-D. After each structure was cooled and shaped, quasi-static compression tests were carried out on HOH1-D to HOH3-D at a compression speed of 5 mm/min, and each sample in this section was tested three times repeatedly. Figure 2 shows the two-dimensional flat structures before deformation, and the specific parameter information regarding each structure is shown in Table 2.

**Figure 3:**
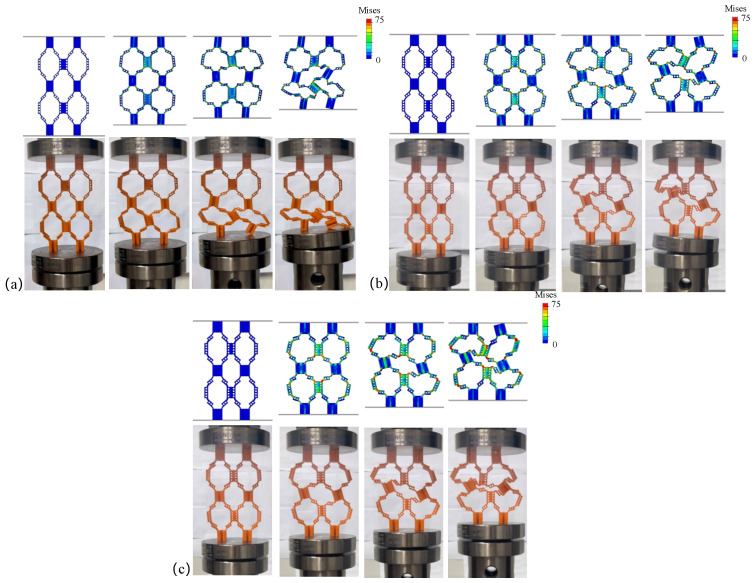
Quasi-static compression process diagram and finite element simulation experiment of hierarchical origami honeycomb structures with different wall thicknesses: (**a**) HOH1-D; (**b**) HOH2-D; (**c**) HOH3-D. Color bars show von Mises stress with values ranging from 0 to 75 MPa.

**Figure 4:**
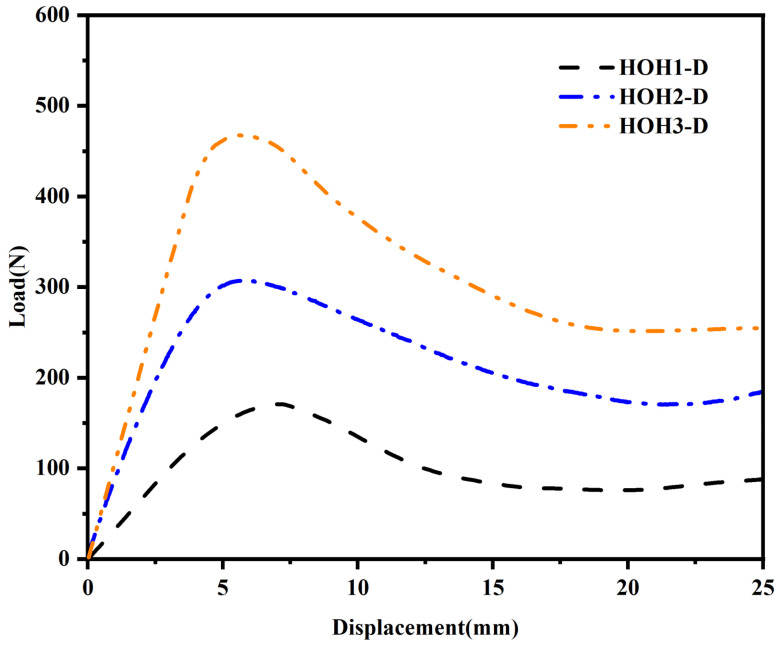
Force–displacement curves of hierarchical origami honeycomb structures with different wall thicknesses in compression tests.

Figure 3a–c show the quasi-static compression process diagrams of structures with different wall thicknesses. Figure 4 displays the average force–displacement curves of three structures with different wall thicknesses. Among them, the deformation forms of HOH2-D and HOH3-D are relatively similar during compression. Before reaching plastic deformation, the unit cells in the upper and lower layers show a tendency to shift outward. With an increase in displacement, the hypotenuses of the two unit cells along the “/” direction are gradually compressed to a collinear state, showing 180°. In contrast, the deformation form of HOH1-D is different. With an increase in compression displacement, the unit cell in contact with the bottom layer first undergoes torsional deformation, and the displacement to reach the peak compression is the largest. This is because the wall thickness is too small, resulting in a greater impact of the hierarchical structure on the overall cell wall. Only changing the wall thickness makes the role of the hierarchical structure smaller as the wall thickness increases. The von Mises stress distribution from finite element simulations (Figure 3) provides further insights into the failure mechanisms. In HOH1-D with the thinnest walls, stress concentrations are primarily located at the hinges connecting different layers, with peak stresses reaching approximately 70–75 MPa at these critical regions. The thin plates themselves experience relatively lower stress levels (30–50 MPa), indicating that failure initiates at the hinge structures. For HOH2-D and HOH3-D with increased wall thickness, the stress distribution becomes more uniform throughout the structure, with both hinges and plates sharing the load more evenly. Peak stress levels remain similar (70–75 MPa), but the volume of material experiencing high stress increases significantly, which contributes to the enhanced energy absorption capacity. The stress analysis confirms that the hierarchical structure’s influence diminishes as wall thickness increases, as the overall structural stiffness becomes dominated by the bulk material properties rather than the geometric design.

Table 3 lists the initial peak forces of the static compression test results of HOH1-D to HOH3-D, and their respective stiffness, specific stiffness, and SEA values are calculated. It is found that with an increase in wall thickness, the initial peak force, stiffness, and SEA value of the structure all increase significantly. Compared to HOH1-D with the thinnest walls, Structure HOH3-D demonstrates substantially improved mechanical performance, with its peak force, stiffness, specific stiffness, and SEA value increased by 187.7%, 267.1%, 87.8%, and 80.7%, respectively. Structure HOH1-D shows the weakest mechanical properties among all tested configurations. It can be seen that the stiffness and initial peak force of the structure change significantly with the change in wall thickness, with nearly threefold improvements observed in stiffness when wall thickness increases from HOH1 to HOH3 parameters.

## 5. Influence of Hinge-to-Thin-Plate-Thickness Ratio Parameter *e* on Compression Performance of Hierarchical Origami Honeycombs

Hierarchical origami honeycomb structures realize shape changes by folding and unfolding along hinges. Observing the compression deformation modes of structures with different wall thicknesses in the previous section, it is found that the first fractured area of each wall thickness model during quasi-static compression is the hinge structure connecting the thin plates. This weak point characteristic of the hinge structure in origami honeycomb structures provides an important research direction for subsequent structural optimization and performance improvement. In this section, the influence of the hinge-to-thin-plate-thickness ratio parameter *e* on hierarchical origami honeycomb structures will be discussed.

**Figure 5:**
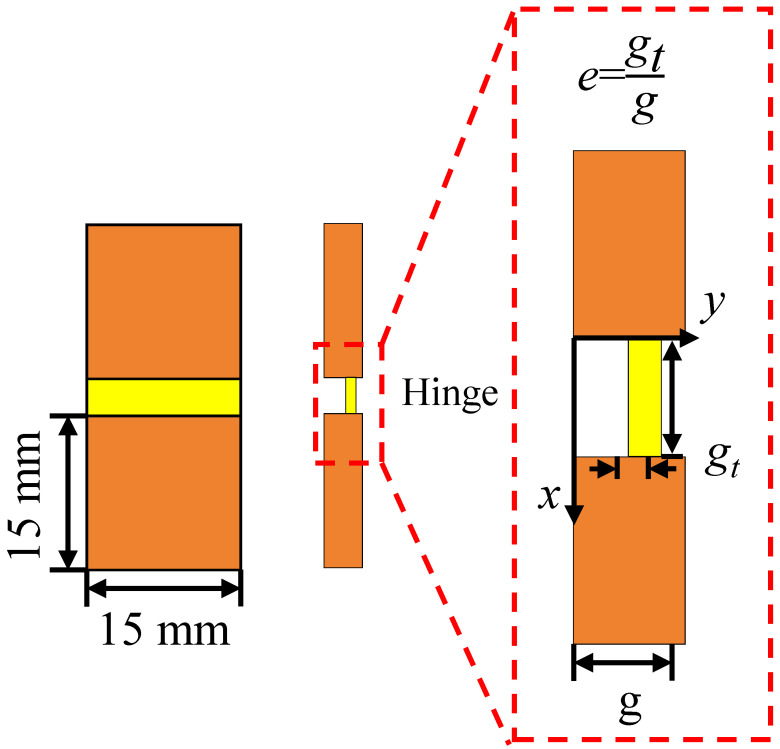
Schematic diagram of geometric parameters of hinge structure.

Hinges are important structures in hierarchical deformable origami honeycombs, which not only participate in deformation but also bear the impact of deformation when compressed. In this section, to improve the strength of the origami structure, the relative thickness of the hinge thickness and the thin plate is adjusted to improve the compressive strength of the hinge. By setting different ratios of hinge thickness to thin plate thickness, the aim is to optimize the performance of the structure to meet specific application requirements. Figure 5 shows a typical origami hinge deformation unit in a hierarchical origami honeycomb, which consists of two rigid thin plates and a deformation hinge. The hinge-to-thin-plate-thickness ratio parameter *e* is defined as

(3) $$e=\frac{g_{t}}{g}$$

Under the condition of keeping the relative density unchanged, the ratio relationship between $g_{t}$ and *g* is changed at the same time to control the change in the *e* value.

To ensure the effective deformation of the thick-plate origami structure and prevent inaccurate deformation caused by material accumulation, the ratio of hinge thickness to thin plate thickness *e* should be controlled within no more than 1/2 [18]. When the *e* value is smaller and the hinge is thinner, the structure can respond faster to thermal stimulation [19]. To further explore the influence of different hinge thicknesses on the compression performance of origami honeycombs, this section designs four hierarchical origami honeycomb structure models with different hinge thicknesses. The selected ratios *e* in this paper include 1/4, 1/3, 5/12, and 1/2, which increase uniformly with a difference of 1/12. On the premise of keeping other structural parameters unchanged, the g value and $g_{t}$ value in the corresponding hierarchical origami honeycomb structure are calculated according to the selected ratios. Based on the above-selected ratios and calculated parameters, the design and preparation of hierarchical origami honeycomb structure models are carried out. The samples are named HD1 to HD3, and the calculated parameters are listed in Table 4. It is worth noting that when the *e* value is 1/3, this configuration corresponds to our previously designed Structure I. Each sample is printed and prepared, and the three-dimensional structures HD1-D to HD3-D are obtained by using the method in the team’s previous research [17]. Each sample is tested three times repeatedly and compared with Structure I-D with an *e* value of 1/3.

**Figure 6:**
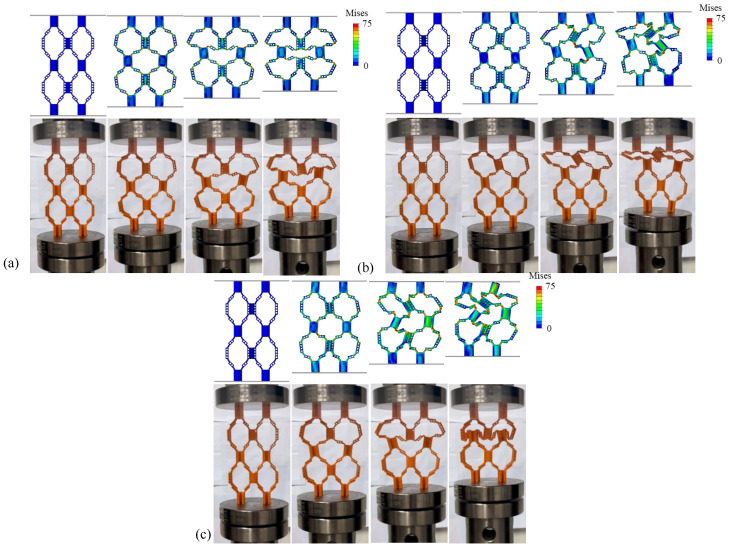
Compression deformation process of hierarchical origami honeycomb structures with 3 different values of e in finite element and experimental methods: (**a**) HD1-D; (**b**) HD2-D; (**c**) HD3-D. Color bars in FEM results show von Mises stress ranging from 0 to 75 MPa.

**Figure 7:**
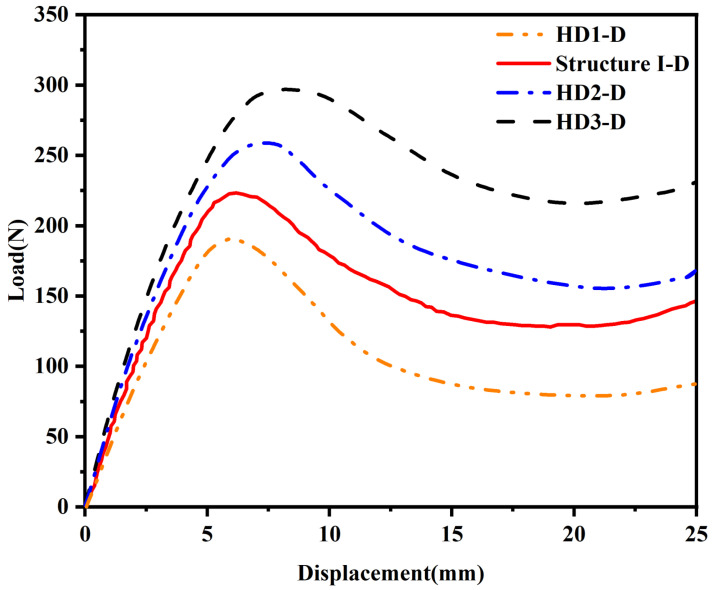
Force–displacement curves of hierarchical origami honeycomb structures with 4 different values of *e* in compression tests.

Figure 6a–c show the static compression processes of structures with different *e* values, and Figure 7 shows the force–displacement curves of samples corresponding to the static compression processes. It can be observed from the figures that with the change in the *e* value, the three samples show differences in compression deformation modes. When the *e* value is 1/4, HD1-D shows a similar deformation failure form to Structure I-D, and the unit cells in the upper and lower layers undergo plastic deformation at the same time. When the *e* values are 5/12 and 1/2, HD2-D and HD3-D first undergo buckling deformation in the unit cell layer close to the indenter or bottom plate at the initial stage of compression. With the continuous increase in compression displacement, the adjacent unit cell layer also undergoes deformation and damage.

**Figure 8:**
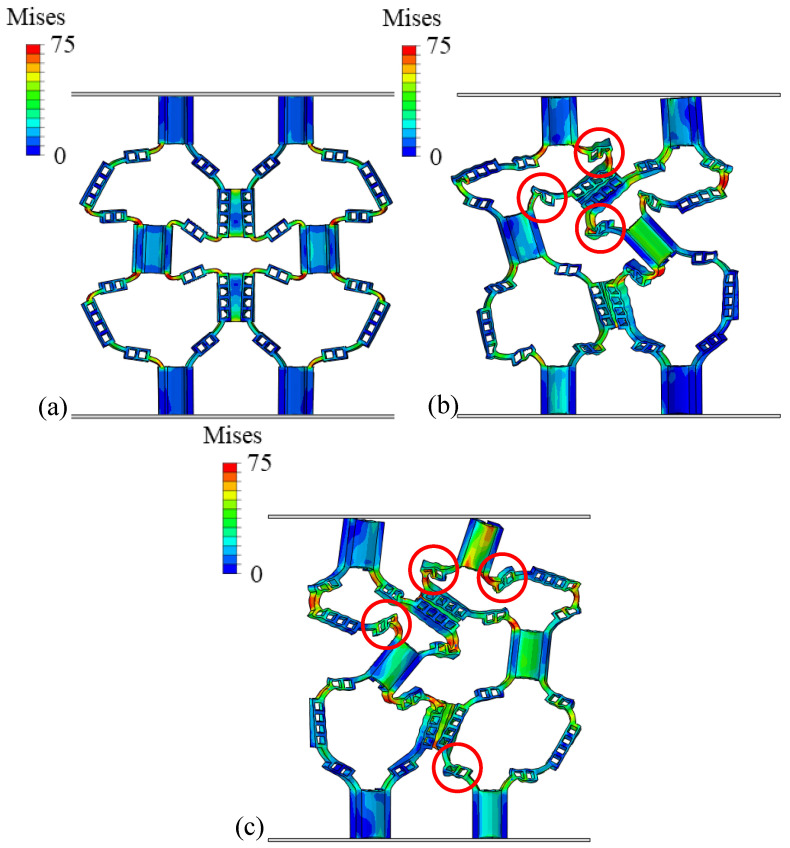
Von Mises stress distribution of each sample after compression: (**a**) HD1-D; (**b**) HD2-D; (**c**) HD3-D. Color bars show stress values ranging from 0 to 75 MPa.

Observing the stress distribution in Figure 8a, further analysis shows that with the increase in hinge thickness, the buckling deformation mechanism of the structure changes significantly. By comparing Structures I-D and HD1-D in Figure 8a, it can be concluded that in structures with thinner hinges, deformation is mainly concentrated in the hinge part, and the thin plate can still maintain good shape integrity under large strain; meanwhile, when the hinge thickness increases, as shown in Figure 8b,c, the buckling deformation of the structure is no longer limited to the hinge area. With the hinge and thin plate thickness gradually increasing, the thin plate part also begins to participate in deformation and damage processes. With the increase in the *e* value, the peak load of the structure increases significantly. In addition, by comparing the force–displacement curves under different *e* values, it can be found that with the increase in the *e* value, the slope of the curve also increases, which indicates that the overall stiffness of the structure is improved. At the same time, the load drop trend after the peak load becomes more gentle, indicating that the ductility of the structure is improved.

**Figure 9:**
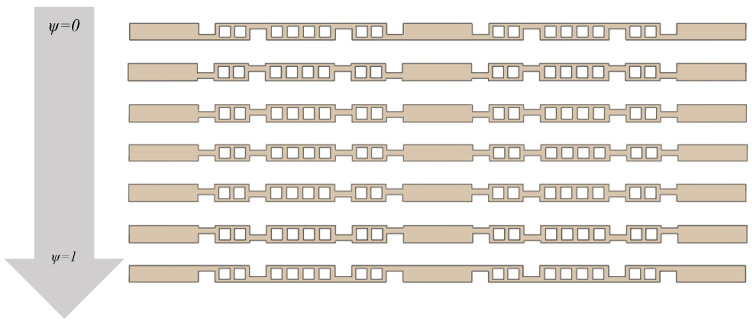
Side view of two-dimensional flat-plate Structures I and JD1 to JD6, where value of ψ changes sequentially from 0 to 1.

The von Mises stress distribution patterns in Figure 9 reveal the underlying mechanisms for these performance differences. In HD1-D with e=1/4 (Figure 8a), stress concentration is highly localized at the hinge regions, with peak stresses of 60–70 MPa confined to narrow zones at the hinge–plate interfaces. The thin plates remain in a relatively low-stress state (below 25 MPa), confirming that deformation is primarily concentrated in the hinges. This localized deformation leads to premature hinge failure and lower overall energy absorption. As the hinge thickness increases in HD2-D (e=5/12, Figure 8b), the stress distribution becomes noticeably more uniform. The hinges still experience peak stresses around 65–75 MPa, but the stressed regions are larger, and the adjacent plates begin to participate in load bearing with stresses reaching 40–50 MPa. For HD3-D with e=1/2 (Figure 8c), the stress distribution is the most uniform, with both hinges and plates experiencing comparable stress levels (50–75 MPa). This more distributed stress pattern allows the entire structure to contribute to energy absorption, explaining the 114% improvement in SEA compared to HD1-D. The stress analysis demonstrates that optimizing the hinge-to-plate-thickness ratio effectively transitions the failure mode from localized hinge fracture to distributed structural deformation, thereby maximizing energy absorption efficiency.

Table 5 lists the initial peak forces of the samples under various *e* values in Figure 7 and their corresponding indenter displacements, and the respective stiffness and SEA values are calculated. The data results show that with the increase in the *e* value, the stiffness, initial peak force, and energy absorption capacity of the samples all show a positive correlation increase. Specifically, when the *e* value increases from 1/4 to 1/2, the initial peak force of the sample increases from 189 N to 297 N, an increase of 57%; the stiffness increases from 3.948 MPa to 4.852 MPa, an increase of 23%; and the energy absorption capacity significantly increases from 0.094 J/g to 0.201 J/g, an increase of 114%, among which the improvement in energy absorption capacity is the most significant.

## 6. Influence of Hinge Position Design Parameter Ψ on Hierarchical Origami Honeycomb Structures

### 6.1. Hinge Position Design Parameter Ψ

**Figure 10:**
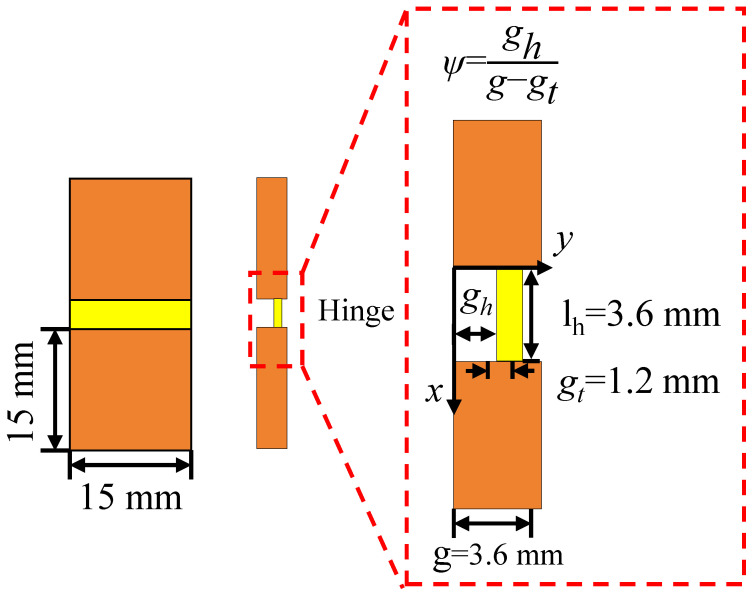
Schematic diagram of geometric parameters of hinge structure.

In this section, the hinge distribution position parameter ψ will be adjusted to demonstrate the high flexibility in the design and manufacturing of 4D-printed deformable hierarchical origami honeycomb structures and analyze their programmable mechanical response characteristics. By adjusting the position of the hinge at the connection of rigid plates, the stiffness of the hierarchical origami honeycomb structure can be systematically adjusted. Figure 10 shows a typical origami hinge in a hierarchical origami honeycomb. The stiffness of the origami hinge is adjusted by changing the hinge position distribution parameter ψ, where ψ is defined as

(4) $$\psi =\frac{g_{h}}{g-g_{t}}$$

where $g_{h}$ and $g_{t}$ represent the position and thickness of the hinge, respectively, and g represents the thickness of the thin plate. The value of $g_{h}$ changes between 0 mm and 2.4 mm with the change in hinge position. When the hinge is aligned with the inner side of the rigid plate, $g_{h}$ is zero, and the ψ value is 0; when the hinge is aligned with the outer side of the rigid plate, the value of $g_{h}$ is equal to $g-g_{t}$, and the ψ value is 1.

### 6.2. Preparation and Experiment of Hierarchical Origami Honeycomb Structures with Different Hinge Position Design Parameters Ψ

In this section, different hierarchical origami honeycomb structures are designed by changing the hinge position design parameters. The selected ψ values for testing are 0.2, 0.4, 0.5, 0.6, 0.8, and 1. The corresponding hierarchical origami honeycomb structures are named JL1 to JL6, respectively. The corresponding two-dimensional flat structures are shown in Figure 9. The deformed structures are obtained using the corresponding simulation method in the team’s previous research [17], and the three-dimensional hierarchical origami honeycomb structures JL1-D to JL6-D under each ψ value parameter are printed and prepared.

**Figure 11:**
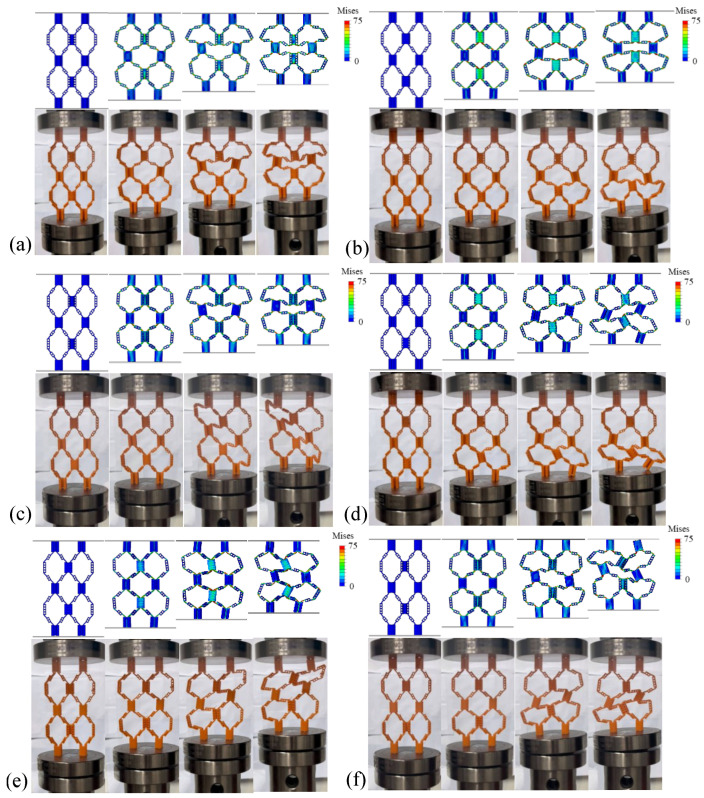
The finite element and experimental compression deformation process of hierarchical origami honeycomb structures corresponding to 6 different values of ψ: (**a**) JD1-D; (**b**) JD2-D; (**c**) JD3-D; (**d**) JD4-D; (**e**) JD5-D; (**f**) JD6-D. The color bars in the FEM results show von Mises stress ranging from 0 to 75 MPa.

**Figure 12:**
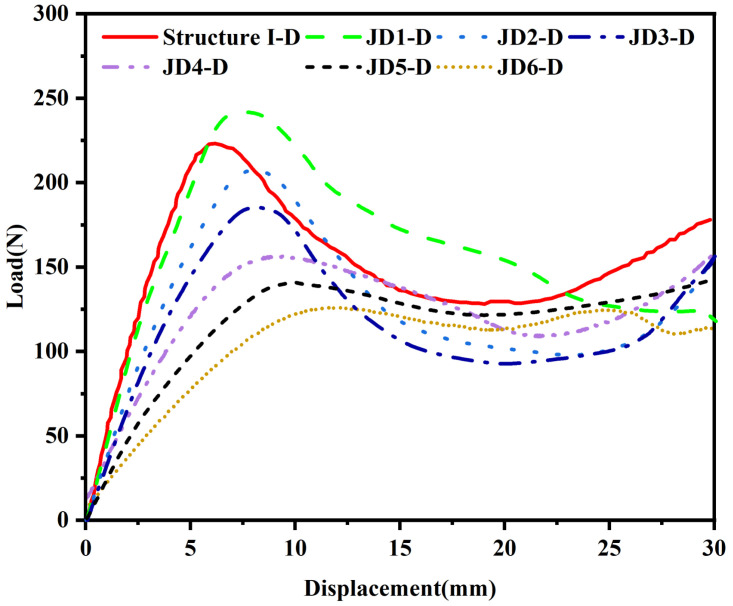
Force–displacement curves corresponding to the static compression processes.

Figure 11a–f describe the static compression processes of structures JD1-D to JD6-D, and Figure 12 shows the force–displacement curves corresponding to the static compression processes. Observing each static compression process, it is found that with the increase in the ψ value, the failure form of the structure gradually changes from inward collapse to outward collapse. This is because the change in hinge position leads to different directions of the force exerted by the indenter on the structure, and the stress point of the structure changes, resulting in different deformation forms. The critical ψ value for the change in form is 0.5. When the ψ value is less than or equal to 0.5, the deformation form of the structure is relatively stable, while when the ψ value is greater than 0.5, the deformation form of the structure becomes asymmetric.

The peak load of structures with different ψ values shows a trend of first increasing and then decreasing. Among them, the JD1-D structure with a ψ value of 0.2 achieves the maximum bearing capacity, and the displacement to reach the peak load is also delayed accordingly. Although the tensile strain of the hinge increases and the compressive strength of the hinge decreases when the ψ value changes from 0 to 0.2, the change in the ψ value changes the connection position between the hinge and the thin plate, thus forming more branch microstructures, making the overall stress distribution of the structure more uniform. From the perspective of the compression deformation process of the structure, the deformation form of the JD1-D structure with a ψ value of 0.2 shows that during the increase in indenter displacement, the two layers of unit cells deform at the same time, and the first failure occurs at the cell wall in the center, i.e., the connection between the upper and lower layers of unit cells. The left and right parts of the entire structure show symmetry, which makes the unit buckling deformation more stable. During the entire compression process, the force value does not drop suddenly, and in the plastic stage after the elastic stage, i.e., the platform stress stage, the load decreases slowly with the increase in strain, which is crucial for improving the energy absorption capacity of the structure. When the ψ value is 0.4, the compression process of the structure shows similar characteristics to that when the ψ value is 0.2, also showing left–right symmetric deformation and failure characteristics. However, when the ψ value is greater than 0.5, the three structures JD-4 to JD-6 tend to be similar in compression performance, but compared with the previous configurations, the failure form of the structure under compression is no longer left–right symmetric but begins to collapse and deform towards one side, resulting in the central unit cell showing a “/” or “\”-shaped deformation. This is because with the increase in the ψ value, the distribution of hinges gradually moves towards the outer side of the thin plate, leading to a change in the stress form of the structure, making the overall stress no longer evenly distributed. Such changes not only affect the stability of the structure but also have a significant impact on its energy absorption characteristics. For configurations with ψ values greater than 0.5, in the platform stress stage, the stress does not change significantly with the increase in axial strain. An analysis of the stress distribution patterns from finite element simulations provides a mechanistic understanding of how ψ influences structural performance. For JD1-D with ψ=0.2, the stress distribution during compression is remarkably symmetric, with peak stresses (65–75 MPa) occurring simultaneously in the hinges of both upper and lower layers. This symmetric stress pattern promotes the stable, simultaneous deformation of multiple layers, contributing to the superior energy absorption observed. The formation of branch microstructures at the hinge–plate connections, resulting from the ψ=0.2 configuration, creates additional load paths that distribute stress more effectively throughout the structure. In contrast, structures with larger ψ values (JD4-D to JD6-D, ψ>0.5) exhibit highly asymmetric stress distributions. Peak stresses become concentrated on one side of the unit cells, typically reaching 70–75 MPa in localized regions, while the opposite side experiences significantly lower stresses (30–40 MPa). This asymmetric loading promotes the one-sided collapse and “/”- or “\”-shaped deformation observed experimentally. The uneven stress distribution not only reduces structural stability but also decreases energy absorption efficiency, as only a portion of the material is effectively utilized during deformation. The stress analysis confirms that the hinge position parameter ψ fundamentally alters the load transfer mechanisms within the hierarchical origami structure, with ψ=0.2 providing the optimal balance between stress distribution and structural stability.

**Figure 13:**
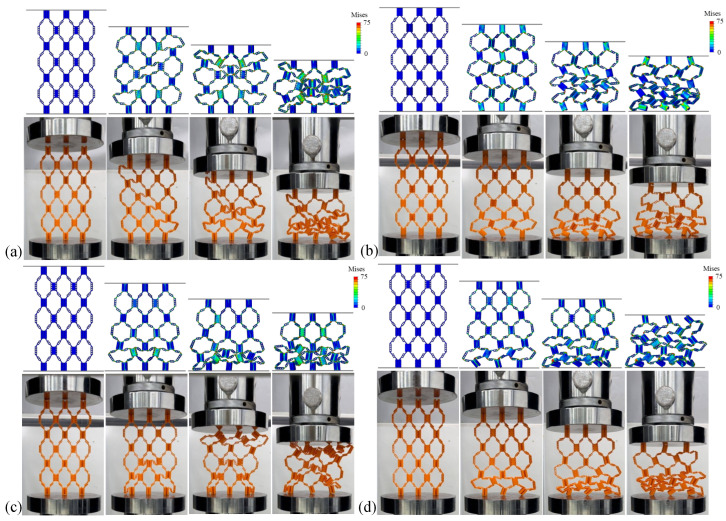
Static compression process diagram and force–displacement curve of hierarchical origami honeycomb structure with different combinations of ψ values: (**a**) ZH1-D; (**b**) ZH2-D; (**c**) ZH3-D; (**d**) ZH4-D. Color bars in FEM results show von Mises stress ranging from 0 to 75 MPa.

Table 6 lists the initial peak forces of each sample in Figure 13 and their corresponding indenter displacements, and the stiffness and SEA values of each sample are calculated. It can be seen from the calculated results that when the ψ value is 0.2, the structure shows the best compression performance, not only having the best peak load but also showing an excellent SEA value, exceeding that of Structure I-D with a ψ value of 0. Its stiffness is slightly lower than that of Structure I-D, but the energy absorption capacity is improved, which is a more beneficial design for an energy-absorbing structure. JD2-D and JD3-D with ψ values of 0.4 and 0.5 have little difference in stiffness and energy absorption, which are weaker than the JD1-D configuration with a ψ value of 0.2. With the increase in the ψ value, the stiffness and energy absorption capacity of the samples decrease significantly.

## 7. Compression Performance of Multi-Layer Hierarchical Origami Honeycomb Structures

To further realize the continuous control of structural stiffness, this section will assign different values to the ψ values of hinges at different layers in the same structure. We will observe the changes in structural strength by combining different ψ values and conduct experimental verification on a larger-scale hierarchical origami honeycomb configuration. This method can not only provide a more refined stiffness adjustment means but also help us deeply understand the influence of different ψ value combinations on structural performance, thus providing a theoretical basis and practical guidance for designing more adaptive energy absorption devices.

### 7.1. Influence of Configuration Expansion on Hierarchical Origami Honeycomb Structures

The hierarchical origami honeycomb structure designed in this paper is composed of the most basic unit cells. Therefore, by stacking unit cells and expanding them in an array form, multi-layer hierarchical origami honeycomb structures are obtained to realize the expansion of structural configurations. In this section, three-layer and four-layer hierarchical origami honeycomb structures are prepared, and their corresponding three-dimensional structures are named TC-D and FC-D, respectively.

**Figure 14:**
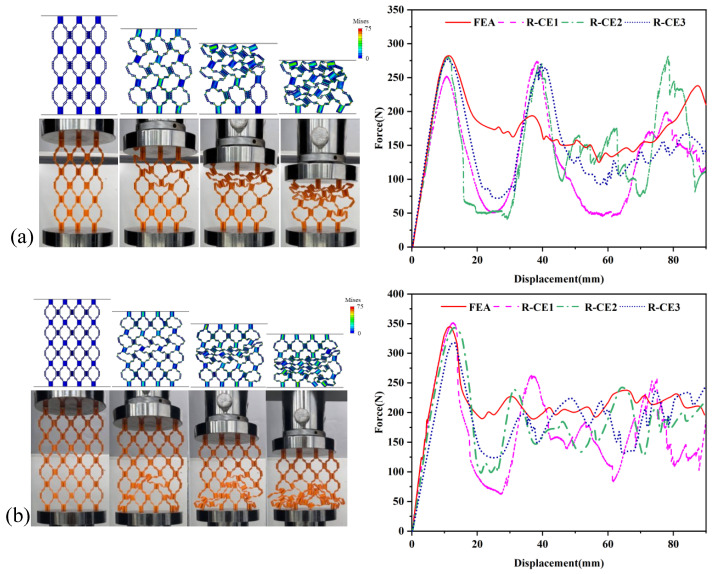
Quasi-static compression process diagram and force–displacement curve of multi-layer hierarchical origami honeycomb structure. (**a**) Three-layer hierarchical origami honeycomb structure. (**b**) Four-layer hierarchical origami honeycomb structure. Color bars in FEM results show von Mises stress ranging from 0 to 75 MPa.

It can be found from the comparison between the simulation results and experiments in Figure 14 that they have a high degree of fitting in the elastic stage. The compression deformation form of the three-layer hierarchical origami structure TC-D first occurs simultaneously in the two layers close to the indenter. After complete crushing, the third layer of unit cells begins to undergo extrusion deformation, so the platform stress stage of the force–displacement curve shows two obvious peaks. The four-layer honeycomb configuration FC-D collapses and deforms along the inverted “V” direction. With the increase in strain, the three layers of unit cells close to the compression bottom plate are crushed one after another. During compression, the shape of the hinge between the bottom unit cells is gradually destroyed, making the cell wall come into contact with the compression bottom plate and be compressed. The initial peak forces and calculated stiffness of the three-layer and four-layer origami honeycomb structures TC-D and FC-D determined by static compression experiments and finite element analysis are listed in Table 7.

It should be noted that while the simulation results show good agreement with experiments in terms of force–displacement curves and load-bearing capacity (Table 7), some discrepancies in deformation modes were observed, particularly for the four-layer configuration FC-D. The experiments exhibited an inverted V-shaped deformation pattern that was not fully captured by the finite element simulation. This discrepancy can be attributed to several factors. First, the 3D-printed samples inevitably contain slight geometric imperfections and layer-by-layer material anisotropy inherent to the FDM printing process, which are not represented in the idealized FE model. These imperfections can trigger preferential buckling in certain layers, leading to asymmetric deformation patterns. Second, minor misalignments of compression platens or friction effects in experiments may introduce loading conditions that differ from the ideal boundary conditions assumed in simulations. Third, multi-layer structures are particularly sensitive to small perturbations due to the presence of multiple competing buckling modes with similar energy levels. Despite these differences in deformation paths, the good agreement in initial peak forces and overall stiffness values (as shown in Table 7) confirms that the simulation approach is effective for predicting the load-bearing capacity and mechanical performance of the hierarchical origami honeycomb structures. The stress evolution during the compression of multi-layer structures reveals complex interactions between layers. For the three-layer configuration TC-D, initial stress concentrations appear in the two layers closest to the indenter, with peak stresses reaching 70–75 MPa. As these layers undergo progressive crushing, stress gradually transfers to the third layer, resulting in the two distinct peaks observed in the force–displacement curve. The four-layer structure FC-D exhibits an inverted V-shaped stress distribution pattern, where the highest stresses (70–75 MPa) develop in the middle layers due to constraint effects from both the top and bottom boundaries. This stress distribution pattern explains the progressive layer-by-layer failure observed in experiments. The finite element stress analysis demonstrates that in multi-layer configurations, the interaction between adjacent layers significantly influences the overall deformation mode and energy absorption characteristics, with stress transfer mechanisms playing a crucial role in determining structural performance.

### 7.2. Multi-Layer Hierarchical Origami Honeycomb Structures with Different Ψ Value Combinations

The 3D printing preparation technology used in this paper gives a high degree of design flexibility to origami honeycomb structures, making it easy to design and manufacture multi-layer hierarchical origami honeycomb structures with different stiffnesses for each layer. The three-layer hierarchical origami honeycomb structure in Section 6.1 is selected, and different ψ values are assigned to each layer. Four three-layer structures with different ψ value combinations are designed, named ZH1-D to ZH4-D, as listed in Table 8, which shows the detailed combination of ψ values for each layer of each structure. The ratio of hinges to thin plates € is 1/3.

**Figure 15:**
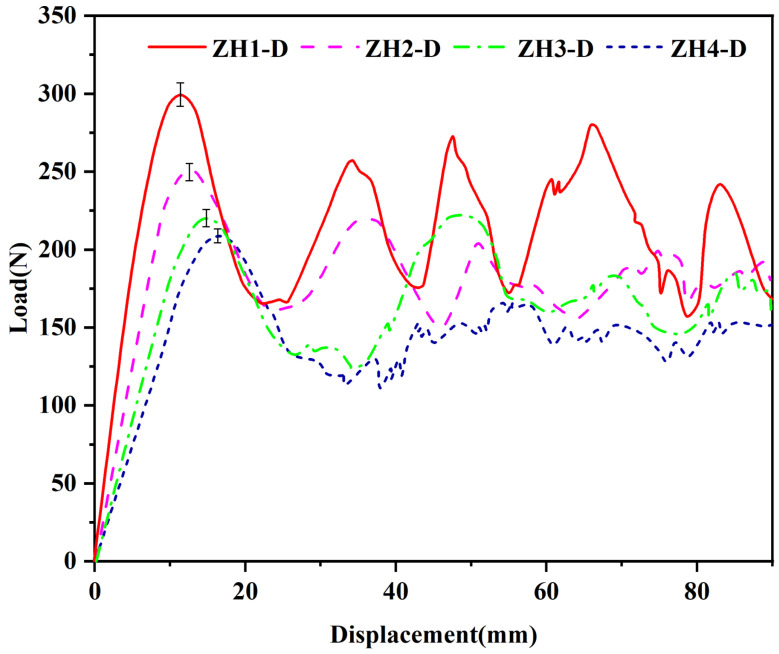
Force–displacement curve of three-layer hierarchical origami honeycomb structure compression test under different combinations of ψ values.

Three quasi-static compression simulation experiments are carried out on each group of samples, and the experimental results are shown in Figure 13. Figure 13a–d show the static compression processes of four samples with different ψ value combinations, and Figure 15 displays the average force–displacement curves of the four samples. The compression deformation of ZH1-D proceeds along the diagonal direction. Since the ψ value of each layer is the same and the smallest (0.2), the stiffness shown by each peak during compression is also similar, and its force–displacement curve is at the top of the four samples, showing the best stiffness in all displacement ranges. However, because the smaller the ψ value, the smaller the bearing capacity, each layer of ZH2-D has a ψ value of 1, which also leads to its force–displacement curve being at the bottom of the four samples, showing the worst stiffness in all displacement ranges. This phenomenon is consistent with the conclusion in Section 4. It is worth noting that ZH3-D presents a unique layer-by-layer compression phenomenon during compression. The ψ values of the first and third layers differ too much, resulting in the third layer structure being crushed first during compression. The first elastic stage on the force–displacement curve is consistent with that of ZH2-D, showing similar stiffness, which indicates that their stiffness is consistent in this stage, and both correspond to the unit cell layer in contact with the bottom plate with a ψ value of 1 that is damaged first. After the third layer is completely crushed, the second layer of unit cells undergoes deformation and failure. However, with the continuation of the compression process, the bearing capacity of ZH3-D gradually exceeds that of ZH2-D, because the stiffness of its second layer (ψ = 0.5) is greater than that of the second layer of ZH2-D (ψ = 1). The ψ values of the second and third layers of ZH4-D are different but relatively close, showing that these two layers of unit cells undergo deformation and failure at the same time when compressed. The stiffness shown in the first elastic stage of its force–displacement curve is greater than that of ZH2-D and ZH3-D but less than that of ZH1-D. With the progress of compression, the subsequent force–displacement curve is similar to that of ZH3-D, fluctuating between the force–displacement curves of ZH1-D and ZH2-D.

The initial peak load, stiffness, and energy dissipation during all stages of the four samples are calculated and summarized in Table 9. ZH1-D shows the best compression performance, with the highest initial peak force, stiffness, and energy dissipation, which are 298 N, 3.273 MPa, and 18.8 J, respectively. ZH2-D shows the worst compression performance, with an initial peak force, stiffness, and energy dissipation of only 209 N, 1.792 MPa, and 12.6 J, respectively. Through a comparative analysis of the compression behavior and force–displacement curves of the four samples, it can be known that the difference in ψ values between different structural layers directly affects the overall stiffness characteristics of the samples. The smallest ψ value in the three layers affects the stiffness and initial peak force in the elastic stage. Samples with the same ψ value distribution in the same layer have similar deformation forms and approximate stiffness performance; the design of ψ values between different layers can effectively regulate the mechanical properties of the samples, and the stiffness characteristics of the samples can be regulated by optimizing the combination of ψ values of each layer.

## 8. Summary

In this paper, through comparative experimental design, the influence of different parameter designs on hierarchical origami honeycombs is studied. First, the influence of wall thickness on the mechanical properties of the structure is clarified. With the increase in wall thickness, the strength of the structure increases positively. The peak force of structures with different wall thicknesses in the elastic stage increases exponentially, and the specific stiffness and SEA value of the structure also increase significantly. Then, parameter optimization comparative experiments are carried out on the hinge part of the structure, including the hinge position distribution parameter ψ and the hinge-to-thin-plate-thickness ratio *e*. For each parameter, three groups of the same samples are printed and prepared for static compression repeated tests at a compression rate of 5 mm/min. The reasons for the influence of each parameter on the structure are analyzed by analyzing the static compression process and average force–displacement curve of each structure. The results show that the structural stiffness decreases gradually with the increase in the ψ value, but when the ψ value is 0.2, the structure shows the optimal initial peak force and energy absorption capacity. The influence of hinge thickness on the strength of the structure is positively correlated. With the *e* value changing from 1/4 to 1/2, various properties of the structure are improved. Then, the mechanical properties of structures with different ψ value combinations in different layers during static compression are verified in the three-layer hierarchical origami honeycomb structure, and multi-level gradient stiffness is realized by this method. By controlling the above structural parameters, the stress–strain curve and SEA value of the hierarchical origami honeycomb structure can be adjusted in a wide range, and this scheme can be used to design and manufacture general origami honeycomb materials.

### Future Examinations

While this study successfully demonstrated the parametric design and optimization of hierarchical origami honeycomb structures, several directions warrant further investigation. First, the development of multimaterial hierarchical origami structures through advanced 4D printing techniques could enable programmable mechanical responses tailored to specific loading scenarios. Second, the integration of smart materials, such as shape memory alloys or piezoelectric materials, into the hinge regions could provide active control over structural stiffness and energy absorption capacity. Third, the exploration of more complex origami patterns beyond the current design, including combinations of different folding motifs, may reveal novel deformation mechanisms and superior energy absorption characteristics. Additionally, dynamic impact testing at various strain rates should be conducted to validate the performance of these structures under realistic crash conditions. Finally, the establishment of a comprehensive theoretical framework linking geometric parameters to mechanical performance would facilitate automated optimization and accelerate the design process for application-specific energy-absorbing devices.

## Figures and Tables

**Table 1:** List of abbreviations and symbols.

Symbol/Abbreviation	Description
PLA	Polylactic Acid
SEA	Specific Energy Absorption
FDM	Fused Deposition Modeling
FEM	Finite Element Method
HOH	Hierarchical Origami Honeycomb
*d*	Hinge thickness (mm)
*g*	Thin plate thickness (mm)
$g_{t}$	Hinge thickness in origami unit (mm)
$g_{h}$	Hinge position distance (mm)
*t*	Wall thickness spacing (mm)
*e*	Hinge-to-thin-plate-thickness ratio, $e=g_{t}/g$
ψ	Hinge position design parameter, $\psi =g_{h}/\left(g-g_{t}\right)$
$l_{h}$	Hinge length (mm)
*K*	Structural stiffness (MPa)
*F*	Applied force (N)
*A*	Cross-sectional area (mm^2^)
ε	Compressive strain
δ	Compression displacement (mm)
*m*	Mass of specimen (g)
*E*	Young’s modulus (GPa)
ν	Poisson’s ratio
$\sigma_{y}$	Yield stress (MPa)
$E_{t}$	Tangent modulus (GPa)

**Table 2:** Design of experimental samples affected by different thicknesses.

Sample	d	g	t	Weight	Printing Time
Structure I	2.4 mm	3.6 mm	0.6 mm	26 g	4.72 h
HOH1	2 mm	3 mm	0.3 mm	19 g	4.08 h
HOH2	2.8 mm	4.2 mm	0.9 mm	32 g	5.35 h
HOH3	3.2 mm	4.8 mm	1.2 mm	37 g	5.92 h

**Table 3:** Static compression test results of HOH1-D to HOH3-D and Structure I-D under different wall thicknesses.

Sample	Initial Peak Force (N)	Stiffness (MPa)	Specific Stiffness (MPa)	SEA (J/g)
HOH1-D	163	2.807	0.148	0.114
HOH2-D	307	6.310	0.197	0.167
HOH3-D	469	10.304	0.278	0.206

**Table 4:** Structural parameters corresponding to different *e* values in Structure I.

Sample	*e*	*g* (mm)	$g_{t}$ (mm)
HD1	1/4(3/12)	3.80	0.95
Structure I	1/3(4/12)	3.60	1.20
HD2	5/12	3.40	1.42
HD3	1/2(6/12)	3.30	1.65

**Table 5:** Static compression test results of samples under different *e* values.

Sample	Displacement *x* (mm)	Initial Peak Force (N)	Stiffness (MPa)	SEA (J/g)
HD1-D	6.1	189	3.948	0.094
Structure I-D	6.9	225	4.155	0.132
HD2-D	7.5	256	4.359	0.159
HD3-D	7.8	297	4.852	0.201

**Table 6:** Static compression test results of samples with different ψ values.

Sample	Displacement *x* (mm)	Initial Peak Force (N)	Stiffness (MPa)	SEA (J/g)
Structure I-D	6.9	225	4.155	0.132
JD1-D	7.8	242	3.954	0.147
JD2-D	8.2	207	3.217	0.113
JD3-D	8.3	186	2.856	0.102
JD4-D	9.2	158	2.054	0.107
JD5-D	10.2	141	1.762	0.099
JD6-D	10.9	126	1.473	0.091

**Table 7:** Initial peak force and corresponding stiffness.

Sample	Initial Peak Force (N)		Stiffness (MPa)	
	**Finite Element**	**Experiment**	**Finite Element**	**Experiment**
Structure I-D	223	225	4.002	4.155
TC-D	281	282	3.843	3.895
FC-D	351	344	3.696	3.714

**Table 8:** Hierarchical origami honeycomb structure with three layers of different combinations of ψ values.

Sample	$\psi_{1}$	$\psi_{2}$	$\psi_{3}$
ZH1-D	0.2	0.2	0.2
ZH2-D	1.0	1.0	1.0
ZH3-D	0.2	0.5	1.0
ZH4-D	0.2	0.5	0.6

**Table 9:** Parameters ψ of three-layer hierarchical origami honeycomb structure.

Sample	Displacement *x* (mm)	Initial Peak Force (N)	Stiffness (MPa)	Energy Dissipation (J)
ZH1-D	11.6	298	3.273	18.9
ZH2-D	15.2	209	1.792	12.6
ZH3-D	14.6	221	1.929	14.5
ZH4-D	12.9	250	2.461	16.1

## Data Availability

The original contributions presented in this study are included in the article. Further inquiries can be directed to the corresponding author.
